# Sympathetic fibre sprouting in the skin contributes to pain-related behaviour in spared nerve injury and cuff models of neuropathic pain

**DOI:** 10.1186/s12990-015-0062-x

**Published:** 2015-09-17

**Authors:** Francisney P. Nascimento, Claire Magnussen, Noosha Yousefpour, Alfredo Ribeiro-da-Silva

**Affiliations:** Department of Pharmacology and Therapeutics, McGill University, McIntyre Medical Building, 3655 Promenade Sir William Osler, Room 1215, Montreal, QC H3G 1Y6 Canada; Alan Edwards Centre for Research on Pain, McGill University, Montreal, QC H3A 0G1 Canada; Department of Anatomy and Cell Biology, McGill University, Montreal, QC H3A 0C7 Canada

**Keywords:** Innervation, NF200, CGRP, Sympathetic nervous system, Neuropathic pain, Sprouting, Skin, Guanethidine, Sympathectomy, Dopamine beta-hydroxylase, Tyrosine hydroxylase

## Abstract

**Background:**

Cuff and spared nerve injury (SNI) in the sciatic territory are widely used to model neuropathic pain. Because nociceptive information is first detected in skin, it is important to understand how alterations in peripheral innervation contribute to pain in each model. Over 16 weeks in male rats, changes in sensory and autonomic innervation of the skin were described after cuff and SNI using immunohistochemistry to label myelinated (neurofilament 200 positive**—**NF200+) and peptidergic (calcitonin gene-related peptide positive**—**CGRP+) primary afferents and sympathetic fibres (dopamine β-hydroxylase positive**—**DBH+)

**Results:**

Cuff and SNI caused an early loss and later reinnervation of NF200 and CGRP fibres in the plantar hind paw skin. In both models, DBH+ fibres sprouted into the upper dermis of the plantar skin 4 and 6 weeks after injury. Despite these similarities, behavioural pain measures were significantly different in each model. Sympathectomy using guanethidine significantly alleviated mechanical allodynia 6 weeks after cuff, when peak sympathetic sprouting was observed, having no effect at 2 weeks, when fibres were absent. In SNI animals, mechanical allodynia in the lateral paw was significantly improved by guanethidine at 2 and 6 weeks, and the development of cold hyperalgesia, which roughly paralleled the appearance of ectopic sympathetic fibres, was alleviated by guanethidine at 6 weeks. Sympathetic fibres did not sprout into the dorsal root ganglia at 2 or 6 weeks, indicating their unimportance to pain behaviour in these two models.

**Conclusions:**

Alterations in sympathetic innervation in the skin represents an important mechanism that contributes to pain in cuff and SNI models of neuropathic pain.

## Background

Animal models are very important to study the mechanisms that contribute to the development and maintenance of neuropathic pain and to identify and assess novel therapeutics to help manage this disabling condition. These models can be broadly divided into two categories, the constriction and transection models. Of relevance to this study are the cuff and spared nerve injury (SNI) models. The cuff model involves placement of a polyethylene cuff of fixed diameter around the sciatic nerve [1], while the SNI model consists of transecting two of the three terminal branches of the sciatic nerve (tibial and common peroneal), leaving the sural nerve intact [2]. Each model produces unique alterations in nociceptive behaviour and in peripheral innervation.

Normally, the skin is densely innervated by primary afferents, including myelinated, neurofilament 200 (NF200) immunoreactive (IR) fibres, most of which transmit non-nociceptive information, and calcitonin gene-related peptide (CGRP)-IR small diameter fibres that convey pain-related information [3]. Injuries to the sciatic nerve often result in an early denervation of the skin, with re-innervation seen at different times depending on the fibre type and model. Significant changes in the density of NF200 and CGRP fibres in the skin have been described in both chronic constriction [4–6] and transection models [6–11], however no studies have looked at the innervation of the skin after cuff.

Nerve injury also provokes changes in the innervation of postganglionic sympathetic fibres, immunoreactive for dopamine β hydroxylase (DBH) and tyrosine hydroxylase (TH). After nerve injury, a sprouting of sympathetic fibres into the dorsal root ganglia (DRG) of rats [12–14] and humans [15] has been reported, where they form baskets around sensory neurons. This abnormal coupling between sensory and sympathetic fibres in the ganglia was initially proposed to account for sympathetically maintained pain, however sympathetic sprouting did not often correlate with the presence or degree of neuropathic pain [16, 17]. Sympathetic fibres also sprout into the upper dermis of the skin, a region from where they are normally absent, after chronic constriction injury (CCI) of the sciatic nerve [4] or the mental nerves in the trigeminal system [18]. Like in the DRG, these newly sprouted sympathetic fibres form close associations with sensory fibres [4, 18]. To date, the contribution of these ectopic sympathetic fibres in the skin to pain, and their presence in other nerve injury models have not been described. This information is important as the extent and rate of sympathetic sprouting varies considerably between models [19].

To fill these gaps in knowledge, we compared the time-dependent changes in peripheral innervation and pain-related behaviour in two distinct and commonly used models of neuropathic pain: the cuff and SNI models. Over 16 weeks, the density of NF200 and CGRP afferents was studied in the skin of the hind paw where mechanical and cold behaviour was measured. In addition, the presence of ectopic sympathetic fibres in the skin and DRG was examined, and their contribution to pain-related behaviour was assessed with guanethidine, a means of chemical sympathectomy, at different times after injury. Understanding how the pattern of skin innervation is altered in neuropathic pain is important, as it is in this region where sensory stimuli, including the nociceptive, are first detected. The results from this paper identify aberrations in sympathetic innervation in the skin as an important mechanism contributing to neuropathic pain.

## Results

### Cuff and SNI rats develop mechanical allodynia and cold hyperalgesia

**Fig. 1:**
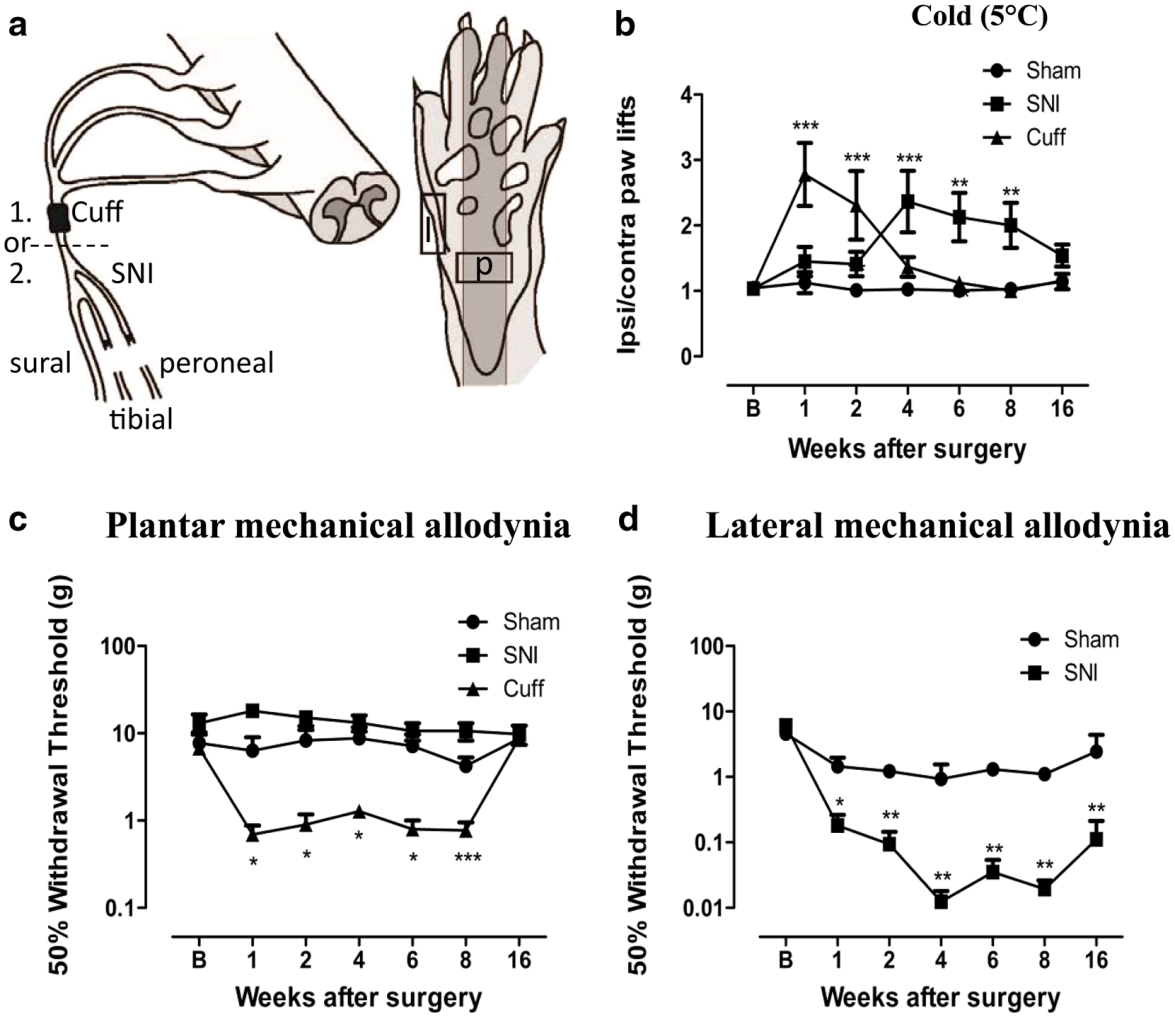
Cuff and SNI rats develop mechanical allodynia and cold hyperalgesia. **a** Illustration of the cuff and SNI models of neuropathic pain and the regions of the paw targeted for behaviour and innervation changes. Animals received either a cuff or a SNI surgery. (1) The cuff model involves the application of a polyethylene cuff around the sciatic nerve before it branches. (2) SNI involves the transection of the tibial and peroneal branches of the sciatic nerve, leaving the sural nerve intact. *Squares* indicate the region of the paw where behaviour was tested and innervation changes measured. Only the plantar paw (*p*) was tested in cuff animals, whereas the lateral paw (*l*), innervated by the spared sural nerve, was also targeted in SNI animals. **b** Responses to cold plate set to 5 °C in cuff, SNI and sham rats. Values represent the paw lift ratio between ipsilateral/contralateral paws. **c** 50 % withdrawal threshold to von Frey fibres in the plantar paw of cuff, SNI and sham rats. **d** 50 % withdrawal threshold to von Frey fibres in the lateral paw of SNI and sham rats. *Each point* represents the mean ± SEM. *p < 0.05, **p < 0.01, ***p < 0.001 compared to sham by two way ANOVA with Bonferroni post hoc test (n = 8 per group)

Spared nerve injury surgery involves the transection of the tibial and common peroneal branches of the sciatic nerve, leaving the sural nerve intact, while cuff surgery involves the application of a polyethylene cuff around the sciatic nerve before it branches (Fig. 1a). The presence of cold hyperalgesia, measured using a cold plate set to 5 °C, and mechanical allodynia, were assessed at 1, 2, 4, 6, 8 and 16 weeks after surgery in cuff and SNI animals. Cuff animals developed early cold hyperalgesia, as indicated by an increase in the ratio of ipsilateral to contralateral paw lifts at 1 and 2 weeks after surgery, with a return to sham levels at later time points (Fig. 1b). The development of cold hyperalgesia was delayed in SNI animals and was present only at 4, 6 and 8 weeks, before returning to normal at 16 weeks (Fig. 1b).

Mechanical thresholds were assessed in the center of the paw, behind the tori, in sham, cuff and SNI rats, in a region we named plantar paw (Fig. 1a; p). Slight ipsilateral hypoalgesia was seen in SNI animals, however these values were not significantly different from sham at any time point. In contrast, application of the cuff resulted in significant reductions in 50 % withdrawal thresholds from 1 to 8 weeks after surgery, with values returning to sham levels by 16 weeks (Fig. 1c). In SNI animals, mechanical thresholds were also measured at the boundary between the hairy and glabrous skin, which remains innervated by the intact sural nerve, in a region we named the lateral paw (Fig. 1a; l). SNI caused a significant reduction in 50 % withdrawal thresholds from 1 to 16 weeks compared to sham animals in the lateral paw (Fig. 1d). Changes in innervation were studied in the same regions of the paw where behaviour was assessed.

### Changes in NF200-IR fibre innervation in the upper dermis of the paw skin of cuff and SNI rats

**Fig. 2:**
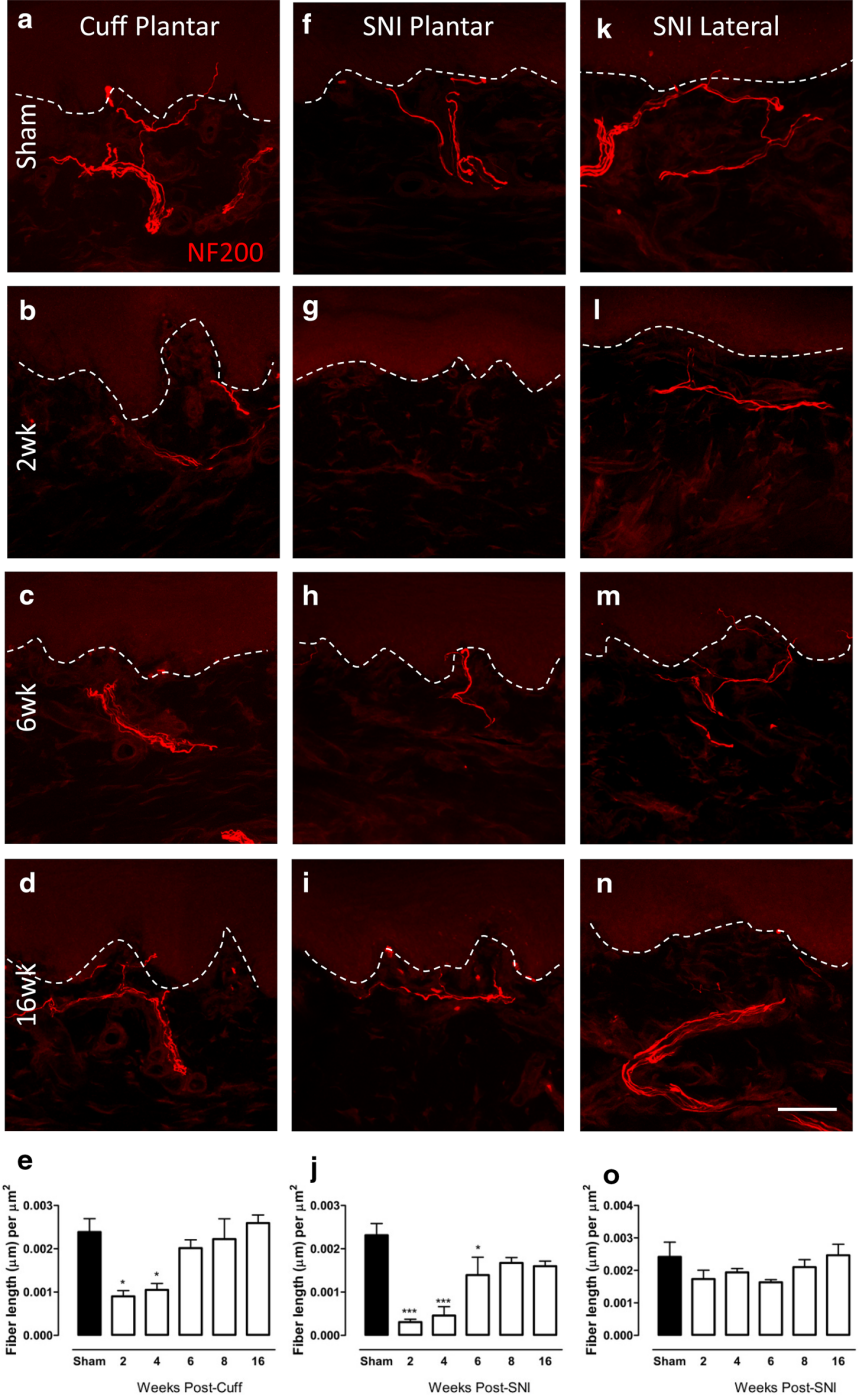
Changes in NF200-IR fibre innervation in the upper dermis of the paw skin of cuff and SNI rats. Photomicrographs show representative examples of NF200-IR fibre innervation (*red*) in the plantar paw skin of sham animals (**a**), and 2 (**b**), 6 (**c**) and 16 weeks (**d**) after cuff. Representative images of NF200-IR innervation in the plantar paw skin of sham animals (**f**) and 2 (**g**), 6 (**h**) and 16 (**i**) weeks after SNI. Representative images of NF200-IR innervation in the lateral paw skin of sham animals (**k**) and 2 (**l**), 6 (**m**) and 16 (**n**) weeks after SNI. *Bar graphs* show average NF200-IR fibre length (µm) per unit area of upper dermis (µm^2^) in the plantar paw skin at various times after cuff (**e**) and SNI (**j**) and in the lateral paw skin after SNI (**o**). *Each point* represents the mean ± SEM (n = 4–6 per group); *p < 0.05, ***p < 0.001 by one way ANOVA with Dunnett’s post hoc; *scale bar* 50 µm

The changes in myelinated fibres, visualized using NF200-immunoreactivity, were examined in the upper dermis of the paw skin at various times after cuff or SNI (Fig. 2). NF200-IR fibres, abundant in the upper dermis of the plantar skin of sham animals, were significantly reduced at 2 and 4 weeks after cuff, and returned to sham levels at the later time points (Fig. 2a–e). SNI produced a more extreme and longer lasting reduction in NF200-IR fibres in the plantar paw skin, with significant decreases in fibre density observed from 2 to 6 weeks, and a recovery to near sham levels at 8 weeks (Fig. 2f–j). No significant changes in NF200 fibre density were observed in the lateral paw after SNI (Fig. 2k–o).

### Changes in CGRP-IR fibre innervation in the upper dermis of the paw skin of cuff and SNI rats

**Fig. 3:**
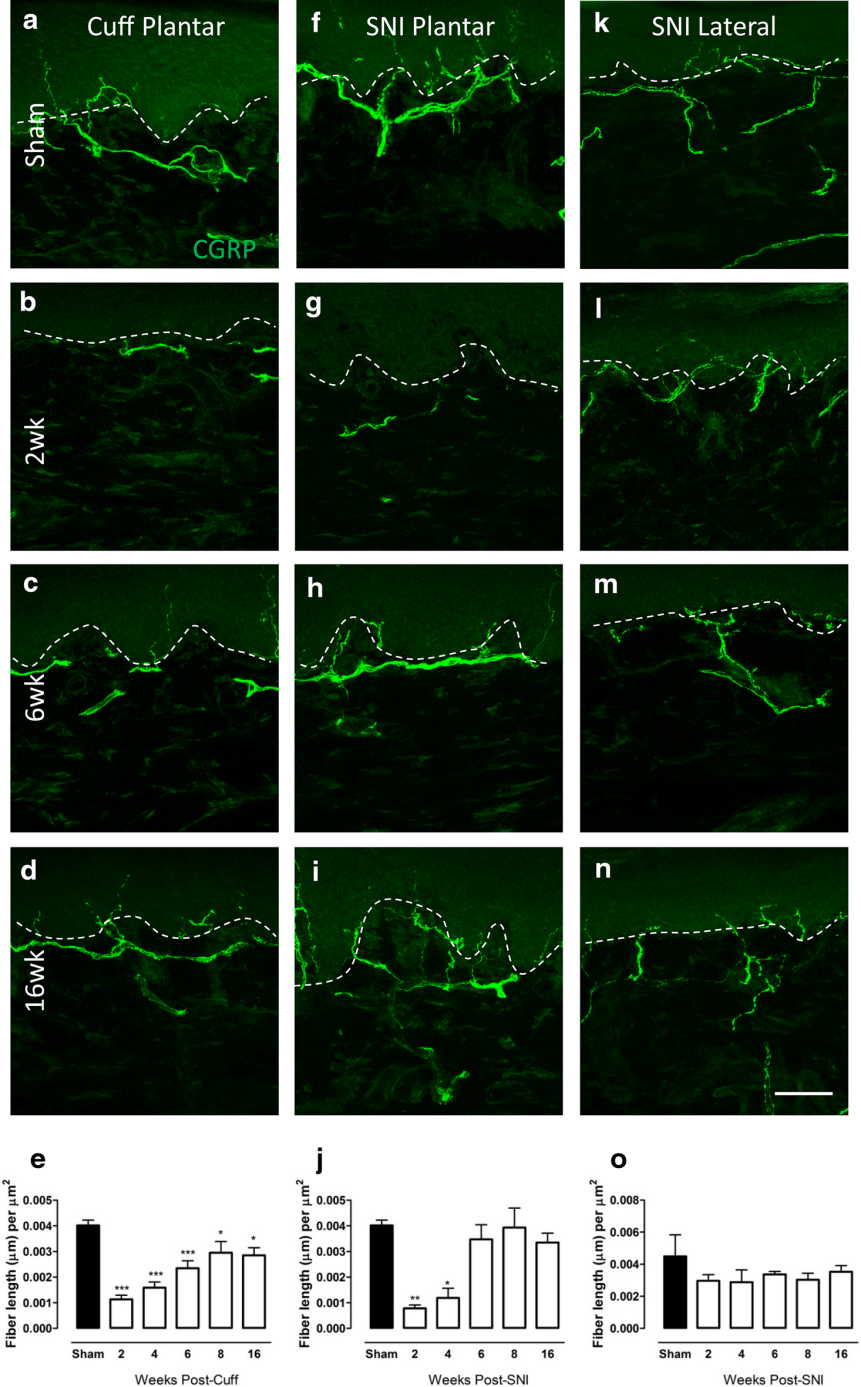
Changes in CGRP-IR fibre innervation in the upper dermis of the paw skin of cuff and SNI rats. Photomicrographs show representative examples of CGRP-IR fibre innervation (*green*) in the plantar paw skin of sham animals (**a**), and 2 (**b**), 6 (**c**) and 16 weeks (**d**) after cuff. Representative images of CGPR-IR innervation in the plantar paw skin of sham animals (**f**) and 2 (**g**), 6 (**h**) and 16 (**i**) weeks after SNI. Representative images of CGRP-IR innervation in the lateral paw skin of sham animals (**k**) and 2 (**l**), 6 (**m**) and 16 (**n**) weeks after SNI. *Bar graphs* show average CGRP-IR fibre length (µm) per unit area of upper dermis (µm^2^) in the plantar paw skin at various times after cuff (**e**), SNI (**j**) and in the lateral paw skin after SNI (**o**). *Each point* represents the mean ± SEM (n = 4–6 per group); *p < 0.05, ***p < 0.001 by one way ANOVA with Dunnett’s post hoc; *scale bar* 50 µm

The changes in peptidergic fibres, visualized using CGRP-immunoreactivity, were examined in the upper dermis of the paw skin at various times after cuff or SNI (Fig. 3). CGRP-IR fibres were abundant in the plantar skin of sham animals and were significantly reduced at all time points studied after cuff. Gradual recovery was seen over time and fibre density approached sham levels by 16 weeks (Fig. 3a–e). While the initial loss of CGRP-IR fibres was more extreme after SNI, fibre density quickly returned to sham levels by 6 weeks (Fig. 3f–j). No significant changes in CGRP fibre density were observed in the lateral paw after SNI (Fig. 3k–o).

### Changes in DBH-IR sympathetic fibre innervation in the upper dermis of the plantar paw skin of cuff and SNI rats

**Fig. 4:**
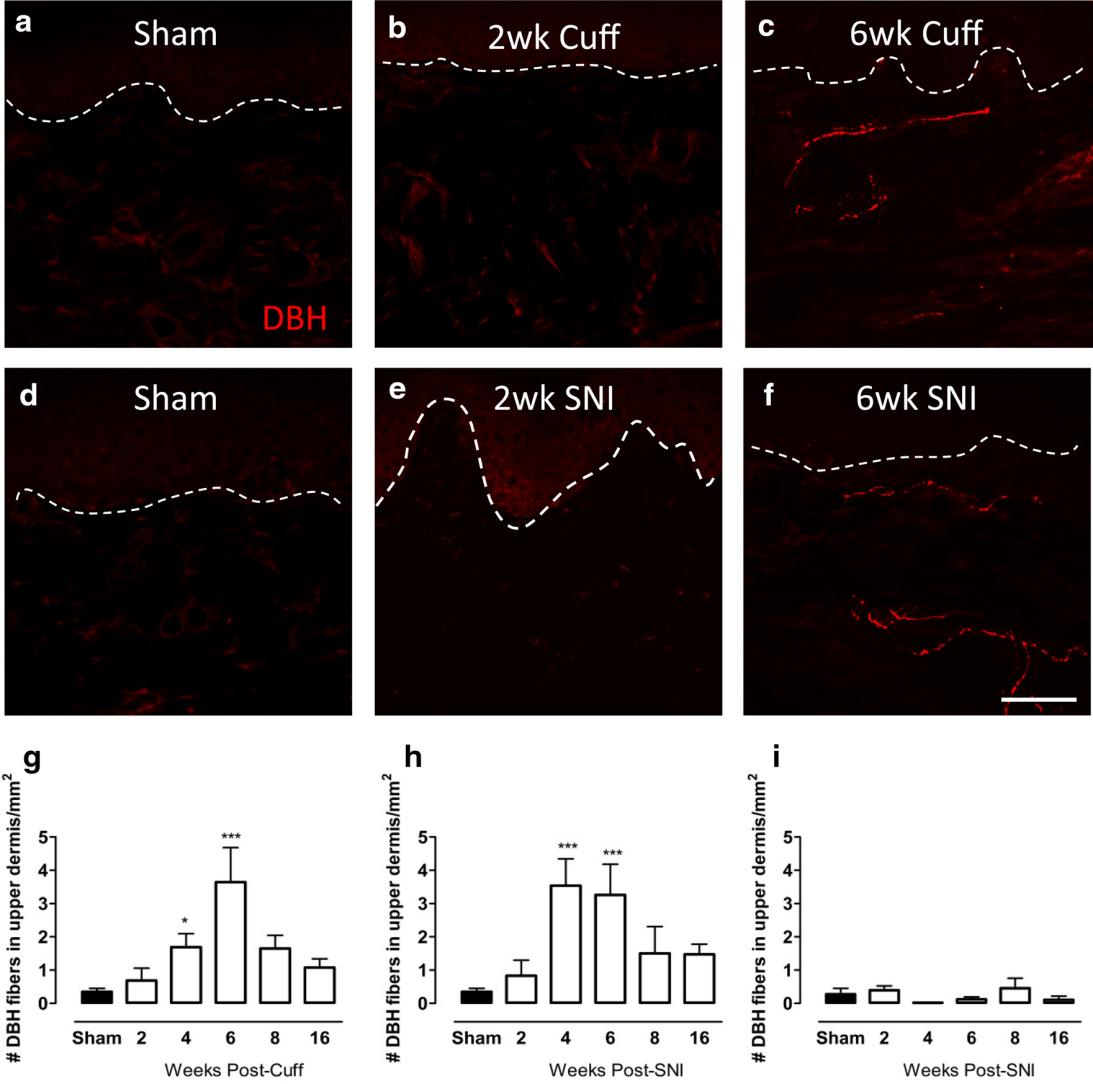
Changes in DBH-IR sympathetic fibre innervation in the upper dermis of the paw skin of cuff and SNI rats. Photomicrographs show representative examples of DBH-IR fibre innervation (*red*) in the plantar paw skin of sham animals and 2, 6 weeks after cuff (**a**–**c**) or SNI (**d**–**f**). *Bar graphs* show average number of ectopic DBH-IR fibres in the upper dermis of the plantar paw skin of cuff (**g**) and SNI rats (**h**) and in the lateral paw skin of SNI animals (**i**) at various times after injury. The values reported are per 1 mm^2^ of upper dermis. *Each point* represents the mean ± SEM (n = 4–6 per group); *p < 0.05, ***p < 0.001 by one way ANOVA with Dunnett’s post hoc; *scale bar* 50 µm

The changes in sympathetic fibres, visualized using DBH-immunoreactivity, were examined in the upper dermis of the paw skin at various times after cuff or SNI (Fig. 4). DBH-IR fibres were almost never observed in the upper dermis of the plantar skin in sham animals, however at 4 and 6 weeks after cuff, they sprouted into this territory and were significantly more abundant (Fig. 4a–c, g). A similar sprouting of sympathetic fibres was observed in SNI animals in the plantar skin, as the mean number of DBH-IR fibres was significantly increased in the upper dermis at 4 and 6 weeks (Fig. 4d–f, h). In both models, the sprouting was only temporary, and while some DBH-IR fibres could still be found in the upper dermis at 8 and 16 weeks, their number was not significantly increased compared to sham. No sympathetic fibres were observed in the upper dermis of the lateral paw skin after SNI (Fig. 4i).

### The effect of chemical sympathectomy with guanethidine on behavioural signs of pain

**Fig. 5:**
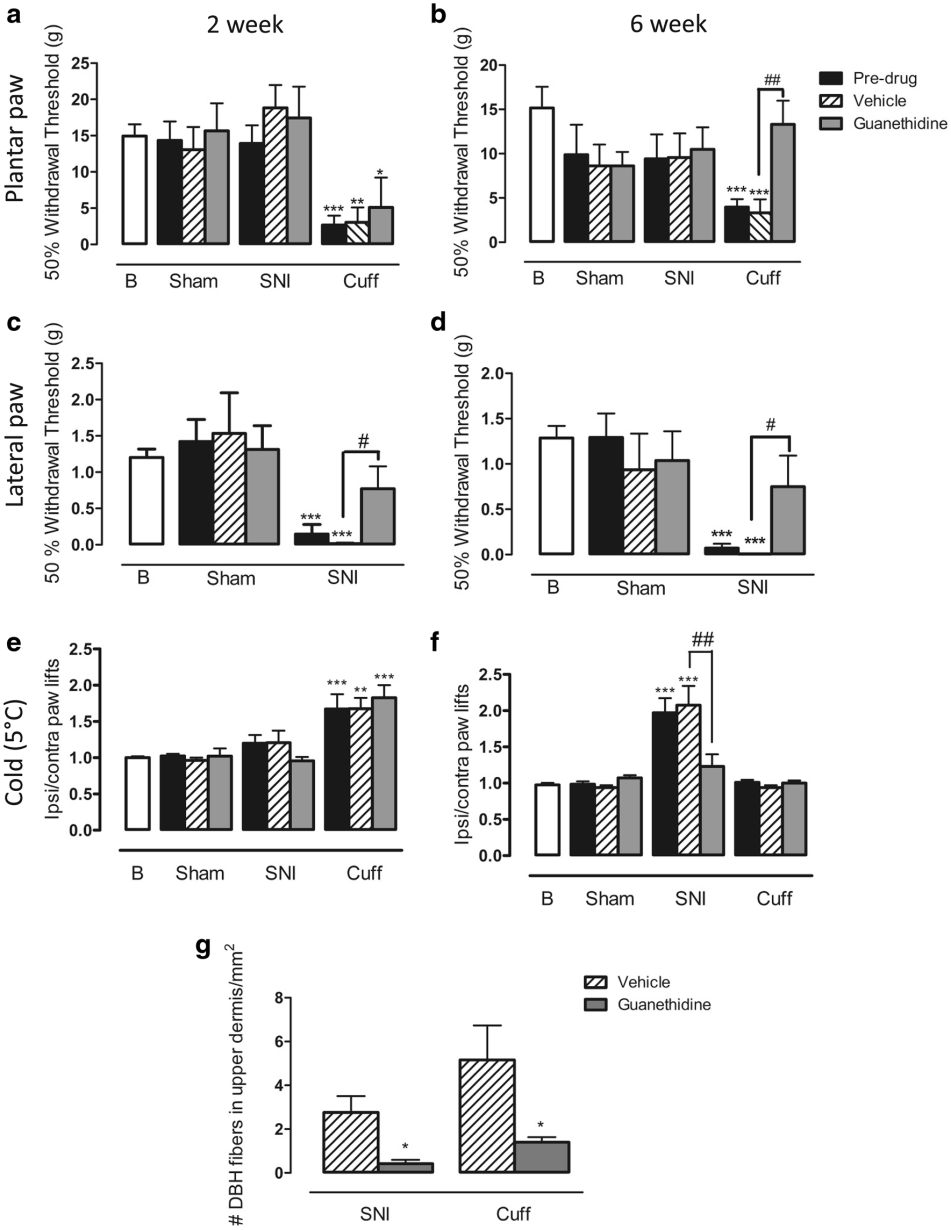
The effect of chemical sympathectomy with guanethidine on behavioural signs of pain. **a** 50 % withdrawal threshold to von Frey fibres in the plantar paw of cuff, SNI, and sham rats treated with guanethidine or vehicle 2 weeks after surgery. Cuff animals had significant mechanical allodynia, and this was unaltered by guanethidine. **b** 50 % withdrawal threshold to von Frey fibres in the plantar paw of cuff, SNI, and sham rats treated with guanethidine or vehicle 6 weeks after surgery. Cuff animals had significant mechanical allodynia which was completely alleviated by guanethidine. **c** 50 % withdrawal thresholds to von Frey fibres in the lateral paw of SNI, and sham rats treated with guanethidine or vehicle 2 weeks after surgery. SNI animals had significant mechanical allodynia which was partially alleviated by guanethidine. **d** 50 % withdrawal thresholds to von Frey fibres in the lateral paw of SNI, and sham rats treated with guanethidine or vehicle 6 weeks after surgery. SNI animals had significant mechanical allodynia which was partially alleviated by guanethidine. **e** Responses to cold plate set to 5 °C in cuff, SNI and sham rats treated with guanethidine or vehicle 2 weeks after surgery. Cuff animals had significant cold hyperalgesia which was unaltered by guanethidine. **f** Responses to cold plate set to 5 °C in cuff, SNI and sham rats treated with guanethidine or vehicle 6 weeks after surgery. SNI animals had significant cold hyperalgesia which was reduced by guanethidine. *Each point* represents the mean ± SEM (n = 6–8 per group). *B* baseline. *p < 0.05, **p < 0.01, ***p < 0.001 compared with baseline, ^#^p < 0.05, ^##^p < 0.01 compared to vehicle treated rats, by a one way ANOVA with Bonferroni post hoc. **g** *Bar graph* showing the mean number of DBH-IR fibres in the upper dermis per 1 mm^2^ in 6 week cuff and SNI animals after guanethidine or vehicle. *p < 0.05 by t-test

To assess the contribution of ectopic sympathetic fibres to pain-like behaviours, a chemical sympathectomy was performed using guanethidine (30 mg/kg) at 2 weeks, when sympathetic fibres were absent from the upper dermis, and at 6 weeks, when they were most abundant in cuff and SNI animals. Two weeks after cuff surgery, animals showed a significant reduction in 50 % withdrawal thresholds in the plantar paw compared with baseline, and these thresholds were unaltered by guanethidine (Fig. 5a). In contrast, 6 weeks after cuff, the significant reduction in 50 % withdrawal threshold observed in cuff animals was completely restored to baseline levels after guanethidine (Fig. 5b). In the lateral paw, 50 % withdrawal thresholds were significantly reduced 2 and 6 weeks after SNI, and guanethidine restored thresholds to baseline values at both time points (Fig. 5c, d). Cold hyperalgesia was present 2 weeks after cuff and was unaltered by guanethidine treatment (Fig. 5e). In SNI animals, cold hyperalgesia was only present at 6 weeks after surgery, and was completely eliminated by guanethidine (Fig. 5f). In all cases, withdrawal thresholds were unaltered in sham animals and guanethidine had no effect. Guanethidine treatment at 6 weeks significantly reduced the number of DBH-IR fibres in the upper dermis of SNI and cuff animals (Fig. 5g).

### Sympathetic fibres do not sprout in the DRG of cuff and SNI animals

**Fig. 6:**
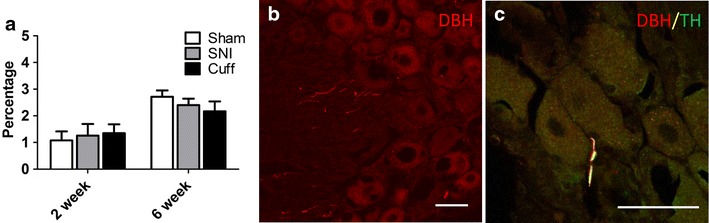
Sympathetic fibres do not sprout in the DRG of cuff and SNI animals. **a** *Bar graph* showing the percentage of cells in close proximity to a DBH-IR sympathetic fibre in the DRG at 2 and 6 weeks after cuff and SNI. **b** Photomicrograph showing representative DBH-IR fibres in the DRG of a 6 week cuff rat. Note that while sympathetic fibres can be seen within the DRG, they are not in close contact with cell bodies and the pattern of staining is no different in sham animals. **c** TH and DBH immunoreactivities were fully colocalized in sympathetic fibres in the DRG; this micrograph shows a rare TH-IR (*green*) + DBH-IR (*red*) sympathetic fibre within the DRG in close proximity to a cell body in a 6 week cuff rat. For quantitative purposes n = 3–4 per group. *Scale bar* 50 µm

Sympathetic fibres have been shown to sprout into the DRG and form baskets around cell bodies in many models of neuropathic pain [12–14, 20–25]. To determine if this occurs in the cuff and SNI models, the percentage of cells in close proximity to DBH-IR fibres was quantified at 2 and 6 weeks after cuff and SNI. The limits of neuronal cell bodies and their nuclei could be easily identified in the sections, in spite of the lack of a specific label for neurons, because of the weak unspecific staining, which was easy to distinguish from the specific fibre labelling (Fig. 6). Sympathetic sprouting in the DRG was not observed either 2 or 6 weeks after cuff or SNI surgery (Fig. 6a). While some sympathetic fibres were seen in the DRG, these were almost never close to cell bodies (Fig. 6b). The pattern of DBH staining in the DRG of cuff and SNI animals was identical to that of sham animals. These findings were confirmed when TH was used as a marker of sympathetic fibres, and sensory neurons were never labeled in the DRG or skin (data not shown). Figure 6c shows an example of one of the very few DBH-IR and TH-IR sympathetic fibres in close proximity to a cell body within the DRG of a 6 week cuff rat.

## Discussion

In the present study, we characterized the time-dependent changes in the innervation of the hind paw and related these to behavioural changes in two commonly used models of neuropathic pain, cuff and SNI. Despite markedly different behavioural responses to evoked stimuli, both models produced a similar early loss and later re-innervation of NF200-IR and CGRP-IR fibres in the upper dermis of the skin. Our primary focus however was on the sympathetic changes in these models and their contribution to pain-related behaviour. We made three observations: (1) ectopic sympathetic fibres sprouted into the upper dermis of the plantar paw skin 4 and 6 weeks after cuff and SNI; (2) these fibres seemed to contribute to cold hyperalgesia in SNI animals, and to the maintenance of mechanical allodynia in cuff animals; (3) sympathetic fibres did not sprout into the DRG in either model.

We focused in the upper dermis of the skin for various reasons. While sensory fibre density is often measured in the epidermis, and is a reliable marker of small fibre neuropathy [26–28], epidermal fibre density does not seem to correlate with neuropathic symptoms [26, 29, 30]. Instead heat and cold pain thresholds correlated best with *dermal* innervation density [31]. Additionally, sympathetic fibres, not normally present in the upper dermis, sprout into this region in neuropathic models [4, 18, 32].

Changes in skin innervation could not alone explain pain-related behaviours. First, although SNI and cuff produced similar early losses of NF200 and CGRP-IR fibres in the plantar paw, at 2 weeks SNI animals were almost significantly hyposensitive to mechanical stimuli and did not have cold hyperalgesia while cuff animals were hypersensitive to both mechanical and cold stimuli. Secondly, despite fibre density being largely unchanged in the lateral paw of SNI animals, prolonged mechanical allodynia was present in this region [2]. After cuff, remaining sensory fibres in the plantar skin may represent intact primary afferents whose sensitization results in altered physiology and nociceptive transmission to the CNS, a finding that has been clearly demonstrated after spinal nerve ligation (SNL) [33–35]. In contrast, in SNI, the few remaining plantar fibres likely originate from spared nerves and do not seem sensitized. Finally, spared Aδ and C fibres from the lateral paw after SNI have been shown to exhibit enhanced activity in response to suprathreshold mechanical stimulation which may account for the prolonged mechanical allodynia present here [36].

In spite of similarities in the time course of re-innervation of CGRP and NF200 fibres, different mechanisms are likely at play in each model. Collateral sprouting from the spared sural and saphenous nerves, which innervate the medial and lateral paw, respectively, probably accounts for re-innervation of the plantar paw in SNI animals [8, 10, 37]. It might be these abnormal fibres that mediate the late onset cold hyperalgesia. Consistent with previous reports [11], we did not see an increase in sensory fibre density in the lateral paw, as fibres sprout from this area to the adjacent denervated paw. Regeneration is virtually impossible in the SNI model, as a 2 mm section is removed from the tibial and peroneal nerves. In the cuff model, considerable re-innervation is probably provided by regeneration of the injured nerves, in addition to some collateral sprouting [32]. In a similar constriction model, primary afferents traced from the injured nerve partially returned to the upper dermis at 4 weeks, demonstrating just how quickly regeneration proceeds [32]. Normalization of innervation coincides with the disappearance of mechanical allodynia in cuff animals.

In addition to alterations in sensory fibre density, SNI and cuff caused an ectopic sprouting of sympathetic fibres in the upper dermis of the plantar skin 4 and 6 weeks after injury. These fibres are assumed to originate from sympathetic fibres innervating blood vessels in the lower dermis, and it is hypothesized that increased target derived nerve growth factor in the denervated area drives this phenomenon [38, 39]. While the time course of sympathetic sprouting closely paralleled what is seen after mental nerve CCI using silk ligatures [18, 32], CCI of the sciatic nerve caused a more robust and prolonged sprouting, persisting until 20 weeks [4]. This may be due to the induction of a more severe inflammatory response initiated by the chromic gut [40, 41]. Despite varying degrees, sympathetic sprouting in the skin is common across many models of neuropathic pain [4, 18, 42].

To better understand how ectopic sympathetic fibres contributed to pain behaviour we performed a chemical sympathectomy at two time points after nerve injury, at 6 weeks, when ectopic sympathetic fibres were present, and at 2 weeks, when they were largely absent. Guanethidine depletes norepinephrine stores in sympathetic terminals [43], and in our hands even eliminated the immunohistochemical detection of sprouted DBH-IR fibres. After cuff, mechanical allodynia was only eliminated by guanethidine when sympathetic fibres were present in the upper dermis, suggesting their likely involvement in the maintenance of allodynia. On the other hand, these fibres seemed to have no importance for cold hyperalgesia after cuff, as hyperalgesia appeared early, and was absent by the time sympathetic sprouting was observed.

Alterations in the sympathetic nervous system play a significant role in pain-related behaviour after SNI. In contrast to the cuff model, there seemed to be a clearer relationship between these ectopic sympathetic fibres and cold hyperalgesia. For example, cold hyperalgesia loosely followed the appearance and disappearance of ectopic sympathetic fibres in the skin of SNI animals and was alleviated by guanethidine at 6 weeks when peak sympathetic fibre sprouting was observed. Because mechanical allodynia was present only in the lateral paw, where ectopic sympathetic fibres were never found, it is suggested that allodynia is driven by other sympathetic changes after SNI. Furthermore, guanethidine significantly alleviated mechanical allodynia at both 2 and 6 weeks, independent of the presence of sprouted sympathetic fibres in the adjacent plantar paw. In SNI, guanethidine’s anti-allodynic effects could alternatively be explained by its ability to reduce plasma norepinephrine [44], whose levels are known to be elevated in some neuropathic pain conditions [45–47]. The results from these experiments point to a complicated relationship between ectopic sympathetic fibres in the skin and pain-related behaviours, and future studies will be required to further unravel their specific contribution to sympathetically maintained neuropathic pain.

Nerve injury has also been shown to provoke a sprouting of sympathetic fibres into the DRG where they formed baskets around sensory neurons [12, 13] and influenced their excitability [24, 25, 48]. Because guanethidine was able to reduce mechanical allodynia 2 weeks after SNI, even in the absence of ectopic sympathetic fibres in the skin, we looked for the presence of sprouted sympathetic fibres in the DRG. DBH fibres were almost never seen in close proximity to cell bodies 2 or 6 weeks after cuff and SNI. Despite its inferiority to DBH, TH, found also in dopaminergic neurons [49] and in a subpopulation of sensory neurons in mice [50], has been extensively used to characterize sympathetic sprouting into the DRG [12, 15, 20, 23, 25, 51]. We were able to confirm that sprouting was equally absent when TH was used to label sympathetic fibres in the DRG. Thus while these fibres may sprout early enough to contribute to SNL induced neuropathic pain, their delayed appearance or even failure to appear in almost all other nerve injury models suggest their relative unimportance to neuropathic pain [17–19, 51, 52]. This is a very interesting finding as sympathetic sprouting into the DRG following nerve injury has been considered one of the major contributors to sympathetically maintained pain [53].

Based on this work, the interaction between sensory fibres and sympathetic fibres is likely more meaningful in the skin, but how do these fibres contribute to pain? We have previously shown a close physical proximity between ectopic sympathetic and C fibres in the skin [4, 18, 32], and we hypothesize that their release of neuroactive compounds sensitizes either the regenerated or sprouted primary afferents. In support of this, adrenergic receptors are upregulated on primary afferents after nerve injury [54, 55] and norepinephrine becomes excitatory for a subset of C nociceptors, enhancing their responsiveness to noxious stimuli [56, 57]. Furthermore, norepinephrine, normally innocuous, produces intense pain when injected into human neuropathic skin [58–60]. Sympathetic fibres also release neuropeptide Y and adenosine triphosphate which could act through Y1, Y2 or P2X3 receptors on primary afferents to modulate their activity [32, 61–66].

One of the most interesting and puzzling findings is that while each model produced a similar sprouting of sympathetic fibres in the skin, these fibres appeared to contribute to different modalities of pain—facilitating the maintenance of mechanical allodynia after cuff and mediating the delayed development of cold hyperalgesia after SNI. There is growing evidence, at least in mice, to support the existence of labelled lines in the pain pathway, such that distinct subsets of primary afferents mediate certain types of pain [67–69]. It is therefore possible that either ectopic sympathetic fibres form closer associations with particular subtypes of primary afferents or that afferent subtypes may differ in their ability to develop adrenergic responsiveness after nerve injury in each model. In each case, only certain fibres would become sensitized by sympathetic mediators. Another interesting observation is the lack of sympathetic nervous system involvement in cold hyperalgesia in cuff animals. This is surprising given its importance in mediating cold hyperalgesia in SNI animals. Thus, the same sensory modality can be caused by different underlying mechanisms depending on the model being used. These findings highlight the important differences between models of neuropathic pain.

## Conclusions

In conclusion, our morphological, behavioural and pharmacological studies show that alterations in sympathetic innervation in the skin represent an important mechanism that contributes to sympathetically maintained pain related behavior in constriction (cuff) and transection (SNI) models of neuropathic pain. We provide evidence for the first time that both models cause ectopic sympathetic sprouting in the skin. Although sympathetic fibres seem to play a role in the pain in both models, there were significant differences suggesting that these models should not be used interchangeably. Finally, we show that the interaction between sensory and sympathetic fibres is most meaningful in the skin, as neither model produced any sympathetic fibre sprouting in the DRG.

## Methods

Adult male Sprague–Dawley rats (200–250 g; Charles River, Canada) were maintained on a 12-h light/dark cycle and allowed access to food and water ad libitum. All protocols were approved by the McGill University Animal Care Committee and followed the guidelines of the Canadian Council on Animal Care and the International Association for the Study of Pain.

### Peripheral nerve lesions

Animals were randomly assigned to receive either a SNI, polyethylene cuff (cuff) or sham operation. All animals were deeply anaesthetized with isoflurane, and were shaved on the left side below the pelvis. The thigh was incised through the skin and then muscle to expose the sciatic nerve and its three terminals branches: the sural, peroneal and tibial nerves. For cuff surgery, the sciatic nerve, before its branching, was isolated from surrounding fascia using a glass probe and a segment of the nerve was elevated to allow placement of a single cuff around the nerve. The cuff consisted of a 2 mm piece of split PE-60 polyethylene tubing with an inner diameter of 0.76 mm (Intramedic PE-60, Fisher Scientific, Canada). For the SNI surgery, the peroneal and tibial nerves were isolated from surrounding fascia and were tightly ligated with 5.0 silk and transected distal to the ligation, removing approximately 2 mm of the distal nerve stump. Care was taken not to manipulate the intact sural nerve. Sham operated rats served as controls, and underwent the same procedure but did not receive any nerve manipulation. In all surgery groups, the muscle and skin layers were closed separately using 4-0 Vicryl absorbable suture (Ethicon, Johnson & Johnson, NJ, USA), and the animals were allowed to recover.

### Behaviour

Animals were tested between 9 AM and 4 PM by blinded experimenters. Following 30 min of habituation to the testing room in their home cages, animals were each placed in a transparent Plexiglas cage atop a wire mesh grid and were allowed to become accustomed to their surroundings for another 30 min before mechanical hypersensitivity was tested. Subsequently, animals were tested for cold hyperalgesia, as described below. Baseline measurements were taken 1 day before surgery, and mechanical allodynia and cold hyperalgesia was assessed 1, 2, 4, 6, 8, and 16 weeks after surgery.

### Mechanical allodynia

Von Frey filaments (0.6, 1, 1.4, 2, 4, 6, 8, 10, 15, 26 g) were applied serially in ascending order of strength to the plantar surface of the hind paw, behind the tori, in cuff, SNI and sham animals with enough force to elicit a slight bend in the filament. Because SNI animals are known to exhibit mechanical allodynia in the lateral paw [2], both SNI and sham animals were tested for mechanical allodynia in this region as well. Each filament was applied for 5 s or until a flexion reflex occurred. An acute withdrawal of the paw was considered a positive response, and signalled the application of the next weaker filament. In the absence of a paw withdrawal response, the next stronger stimulus was presented. After the first positive filament, four additional filaments were applied and the 50 % withdrawal threshold was calculated using the methods outlined by Chaplan et al. [70]. Mechanical allodynia was considered as a significant reduction in withdrawal threshold when compared to shams, as measured by a two way analysis of variance (ANOVA) with Bonferroni post hoc. Statistical significance was set at p < 0.05.

### Cold hyperalgesia

To assess the presence of cold hyperalgesia after nerve injury, animals were placed on a cold plate (Cold and Hot Plate Test, Bioseb) for 5 min, in which the platform was set to a temperature of 5 °C. The first minute was considered habituation, and the total number of paw lifts (including steps, paw lifts and licks) were recorded for each paw from 1 to 5 min, as adapted from Jasmin et al. [71]. The ratio of ipsilateral (left) to contralateral (right) paw lifts was calculated for each animal. Cold hyperalgesia was considered as a significant increase in the ratio of paw lifts when compared to sham animals as measured by two-way ANOVA with Bonferroni post hoc with p < 0.05 considered significant.

### Drug administration to suppress sympathetic fibre function

To assess the contribution of the sympathetic nervous system to mechanical allodynia and cold hyperalgesia, chemical sympathectomy was performed using guanethidine. Following behaviour testing at 2 or 6 weeks after surgery, two intraperitoneal injections of guanethidine sulfate (30 mg/kg; Santa Cruz Biotechnology) or vehicle (saline 1 ml/kg) were given 24 h apart [72]. Behaviour was assessed 4 h after the second injection. The following groups were used: sham + vehicle, sham + guanethidine, SNI + vehicle, SNI + guanethidine, cuff + vehicle and cuff + guanethidine. Pre-drug values (SNI + vehicle, SNI + guanethidine; cuff + vehicle, cuff + guanethidine) were pooled together since no statistically significant difference was observed. For each group of animals, pre and post drug values were compared with baseline values using a one way ANOVA with Bonferroni post hoc with p < 0.05 considered significant.

### Animal perfusion and histological processing

Cohorts of animals were sacrificed at 2, 4, 6 and 8 weeks post-surgery. Animals from the behaviour time course were used for the 16 week time point. Animals used for the guanethidine experiment were perfused at 2 and 6 weeks, following the final behaviour testing to assess the effect of guanethidine on sympathetic fibres. In all cases, rats were deeply anesthetized with Equithesin (0.3 mL/100 g) and transcardially perfused with 100 mL of perfusion buffer followed by 500 mL of 3 % paraformaldehyde and 15 % saturated picric acid (v/v) in 0.1 M phosphate buffer (PB), pH 7.4, for 30 min. The glabrous skin from the left hind paw, specifically the skin just behind the tori, and the lateral paw skin, both regions which were targeted during the behaviour testing, and the L4 ipsilateral DRG were removed, postfixed by immersion for 1 h in the above fixative and cryoprotected in 30 % sucrose in PB for 24 h at 4 °C. Tissue was embedded in an optimum cutting temperature medium (Tissue Tek, OCT) and 50 and 14 µm thick sections of skin and DRG, respectively, were cut on a cryostat (Leica, Germany) at −20 °C. Skin sections were collected as free-floating and DRG sections were slide mounted directly.

### Immunohistochemistry

To determine the changes in innervation after SNI and cuff, skin sections were processed for immunohistochemistry using antibodies against NF200, CGRP and DBH to label myelinated fibres, peptidergic sensory fibres and sympathetic fibres, respectively. DRG sections were labelled with DBH and TH. Free-floating and slide staining were performed using the same protocol. All sections were washed for 30 min with PBS containing 0.2 % Triton-X (PBS-T), incubated in 50 % ethanol for 30 min and washed in PBS-T. Depending on the species in which the secondary antibody was raised in, the tissue was either pre-incubated in 10 % normal donkey or normal goat serum for 1 h to block unspecific staining. Primary antibodies were used at the following concentrations—anti-NF200 (1:5, mouse monoclonal, Abcam), anti-CGRP (1:2000, rabbit polyclonal, Sigma), anti-DBH (1:50, mouse monoclonal, Medimabs) and anti-TH (1:2000, rabbit polyclonal, Millipore) made in PBS-T and incubated overnight on a shaker at 4 °C. Following 30 min of washes with PBS-T, the tissue was incubated with the appropriate secondary antibody diluted in PBS-T for 2 h at room temperature—goat anti-rabbit conjugated to Alexa Fluor 488 (1:800, Molecular Probes), goat anti-mouse conjugated to Alexa Fluor 568 (1:800, Molecular Probes), or donkey anti-mouse conjugated to Rhodamine Red X (1:200, Jackson Immunoresearch). Following 30 min of washes, free-floating skin sections were mounted on gelatin-subbed slides and all slides were coverslipped with Aqua Polymount (Polysciences).

Representative images were taken using a Zeiss LSM510 confocal microscope equipped with AR and He–Ne lasers using a 40× water-immersion objective. Z stacks of confocal optical sections were obtained, exported as TIFF files and adjusted using Adobe Photoshop for brightness and contrast only.

### Quantification of fibre density

Images used for quantification were taken on a Zeiss Axioplan 2e imaging fluorescence microscope (Carl Zeiss Canada, Toronto), with a 40× objective. Images were acquired with a high-resolution color digital camera with Zeiss Axiovision 4.8 software. All of the quantification was done by a blinded experimenter. Changes in NF200 and CGRP innervation in the paw skin were determined by analyzing the density of fibres within the upper dermis, defined as the area 150 µm from the dermal-epidermal junction [4]. Four randomly chosen fields per skin section, measuring 312.4 µm × 250.6 µm each, from 6 sections were captured, totaling 24 images per animal; 4–6 animals were used for each time point. Quantification was performed using an MCID Elite image analysis system (Imaging Research Inc.) to determine the total fibre length (µm) per unit area of upper dermis (µm^2^), as described by us previously [5]. Briefly, we used a function of the MCID software that was developed to specifically and accurately measure fibres. After detection, fibres were skeletonized to 1 pixel in width, and the total fibre length per unit area was determined and compared using an ANOVA with Dunnett’s post hoc, with p < 0.05 considered significant.

### Quantification of DBH fibres

Sympathetic fibre density was determined by counting the number of DBH-IR fibres in the upper dermis, defined as the area spanning 150 µm below the dermal epidermal junction. This region was chosen as sympathetic fibres are normally absent from this area in sham animals [4]. The value for the total area analyzed was calculated by multiplying the total length of the section by the thickness of the upper dermis (150 µm). The total number of DBH-IR fibres in the upper dermis across six sections per animal was counted. The mean number of fibres in the upper dermis per unit area (normalized to be 1 mm^2^) was compared using an ANOVA with Dunnett’s post hoc, with p < 0.05 considered significant.

In the DRG, the percentage of cell bodies in close proximity to a sympathetic fibre was quantified at 2 and 6 weeks after cuff, SNI and sham surgery. To do this, the total number of cell bodies in close proximity (defined as being directly adjacent) to a sympathetic fibre was divided by the total number of cell bodies in each section, using images taken with the fluorescence microscope. In both instances, only cells with visible nuclei were counted. Quantification was performed in 10 DRG sections per animal.
